# Effects of Transcutaneous Vagus Nerve Stimulation on Gastrointestinal Symptoms and Cardiovascular Autonomic Outcomes: A Systematic Review and Meta-Analysis

**DOI:** 10.3390/neurolint18070127

**Published:** 2026-07-03

**Authors:** María Pérez-Montalbán, Encarna García-Domínguez, Manuel Pabón-Carrasco, Ángel Oliva-Pascual-Vaca

**Affiliations:** 1 Department of Physiotherapy, Faculty of Nursing, Physiotherapy and Podiatry, University of Sevilla, 41009 Sevilla, Spain; 2 Digestive System Department, University Hospital of Puerto Real, 11510 Cádiz, Spain; 3 PAIDI-CTS-1050 Research Group “Complex Care, Chronicity and Health Outcomes”, Department of Nursing, Faculty of Nursing, Physiotherapy and Podiatry, University of Sevilla, 41009 Sevilla, Spain; 4 Institute of Biomedicine of Seville-IBiS (Virgen del Rocío and Macarena University Hospitals/CSIC/University of Sevilla), 41013 Sevilla, Spain; 5 Madrid Osteopathic School, 28033 Madrid, Spain

**Keywords:** transcutaneous vagus nerve stimulation, autonomic nervous system, neuromodulation, cardiac autonomic neuropathy, brain–gut axis, gastrointestinal disorders

## Abstract

**Background**: Autonomic dysfunction is increasingly recognized as a key mechanism in disorders involving the brain–gut axis and gastrointestinal symptom generation. Transcutaneous vagus nerve stimulation (tVNS) is a noninvasive neuromodulatory technique investigated for its potential effects on autonomic regulation. **Methods**: A systematic review and meta-analysis were conducted following PRISMA guidelines, with protocol registration in PROSPERO. Randomized controlled trials (RCTs) investigating auricular or cervical tVNS in patients with visceral disorders were included. Continuous outcomes were pooled using mean differences (MDs) or standardized mean differences (SMDs) with 95% confidence intervals (CIs). When multiple publications originated from the same trial population, only independent datasets were considered for quantitative synthesis to avoid double-counting participants. Risk of bias was assessed using the PEDro scale and certainty of evidence with the GRADE approach. **Results**: Seven RCTs met the inclusion criteria. tVNS demonstrated a small but statistically significant improvement in gastrointestinal symptoms based on change-from-baseline GSRS scores (MD −0.19; 95% CI −0.29 to −0.09; *p* < 0.001; I^2^ = 0%). Although statistically significant, the magnitude of this effect was modest, and its clinical relevance remains uncertain. No significant effects were observed on cardiac vagal tone (SMD 0.19; 95% CI −0.53 to 0.90; *p* = 0.61; I^2^ = 73%) or systolic blood pressure (MD −1.24 mmHg; 95% CI −6.69 to 4.21; *p* = 0.66; I^2^ = 0%). Evidence regarding cardiac autonomic neuropathy (CAN) was limited to a single independent randomized controlled trial, which found no significant differences between tVNS and sham stimulation. **Conclusions**: tVNS provides modest statistically significant improvements in gastrointestinal symptoms, supporting its role as a symptomatic neuromodulatory intervention. However, the available evidence for this outcome was based on only two studies, and the clinical relevance of the observed effect remains uncertain. No statistically significant pooled effects were observed for the cardiovascular autonomic markers assessed in this review. Evidence regarding CAN was limited to a single independent study. Therefore, the available evidence remains limited and heterogeneous, and further high-quality randomized controlled trials are warranted.

## 1. Introduction

Owing to their high prevalence, chronicity, and limited response to conventional therapeutic strategies, functional dyspepsia, irritable bowel syndrome, pancreatitis, and other disorders of the abdominal viscera represent a significant global clinical burden [1,2,3]. Furthermore, affected individuals consistently report markedly diminished quality of life compared with the general population [4,5].

The pathophysiology of these gastrointestinal disorders is complex and may involve bidirectional dysregulation of the gut–brain interaction (via the gut–brain axis), as well as microbial dysbiosis, altered mucosal immune function, visceral hypersensitivity, and abnormalities in gastrointestinal motility [6]. The so-called brain–gut axis relies on an intricate network comprising the vagus nerve (VN), together with sympathetic, endocrine, and immune connections [7]. This axis is becoming increasingly important as a therapeutic target for gastrointestinal disorders. Moreover, interventions at this level have been shown to confer benefit in mood and anxiety symptoms, which are highly prevalent among these patients [8,9,10].

The VN (cranial nerve X) originates in the medulla oblongata and emerges from the skull through the middle compartment of the jugular foramen [11,12]. It is a mixed cranial nerve, composed primarily of unmyelinated fibers, with approximately 80% afferent and 20% efferent components [13]. In the cervical region, it provides motor innervation to most pharyngeal and laryngeal muscles, which mediate deglutition and phonation. Within the thoracic cavity, it conveys parasympathetic efferent fibers to the cardiac plexus. In the abdominal region, vagal fibers regulate gastrointestinal smooth muscle contractility and motor activity [8].

Notably, abdominal vagal afferents encompass mucosal mechanoreceptors, chemoreceptors, and tension receptors within the esophagus, stomach, and proximal small intestine, as well as sensory endings in the liver and pancreas [8] that convey visceral, somatic, and gustatory information. Abdominal vagal efferents, in turn, contribute to the regulation of gastrointestinal motility and secretory activity [13]. Through these fibers, the VN plays a modulatory anti-inflammatory role. The anti-inflammatory properties of vagal afferents were first described by Harris in 1950 in relation to the hypothalamic–pituitary–adrenal axis [14,15]. The anti-inflammatory properties of vagal efferents were subsequently characterized by Tracey and colleagues in 2000, who defined this mechanism as the ‘cholinergic anti-inflammatory pathway’ [16,17].

Dysregulation of vagal neural activity may lead to clinical disorders. Vagus nerve stimulation (VNS) has been proposed as a novel nonpharmacological analgesic intervention for pain management [13]. This technique operates by delivering electrical impulses to the nerve and can be administered via two distinct approaches: (i) direct invasive stimulation, which requires surgical implantation of a pulse generator, and (ii) indirect noninvasive transcutaneous stimulation, which targets auricular or cervical branches of the vagus nerve through the skin [8,18,19]. In healthy subjects, it has previously been demonstrated that VNS, achieved through slow, deep breathing, can prevent central sensitization in a validated model of esophageal pain hypersensitivity [20].

In recent years, multiple studies have investigated the efficacy of transcutaneous vagus nerve stimulation (tVNS) in patients with abdominal visceral disorders [21,22,23,24]; however, the findings have been heterogeneous and, at times, contradictory. Given the increasing interest in noninvasive neuromodulatory approaches, critically synthesizing the available evidence is essential. Accordingly, the present systematic review and meta-analysis aimed to evaluate the effectiveness of tVNS in individuals with peridiaphragmatic abdominal visceral disorders, providing a comprehensive perspective on its therapeutic potential and informing future clinical research.

## 2. Materials and Methods

A systematic review with meta-analysis was conducted according to the Preferred Reporting Items for Systematic Reviews and Meta-Analyses (PRISMA) [25]. The completed PRISMA checklist is provided in the Supplementary Materials (Supplementary Figure S1). This review was previously registered in the International Prospective Register of Systematic Reviews (PROSPERO) under the following number: CRD420251077085.

### 2.1. Data Source and Search Strategy

Two authors (M.P.-M. and A.O.-P.-V.) independently performed a literature search in PubMed, Scopus and Web of Science, which was completed in July 2025. The authors examined the reference lists from the retrieved full-text studies. Concerns regarding the search strategy were raised with a third author (E.G-D.). According to Medical Subject Headings (MeSH), the keywords employed in our search were “transcutaneous vagal nerve stimulation”, “noninvasive vagal nerve stimulation”, “transcutaneous auricular vagal nerve stimulation”, “peridiaphragmatic abdominal viscera disorder”, “functional dyspepsia”, “gastroesophageal reflux”, “hiatal hernia”, “primary sclerosing cholangitis”, “pancreatitis”, “gastritis”, “duodenitis”, “diabetes”, and “esophageal stenosis”. For each database, a specific keyword combination was employed using the appropriate tags and the Boolean operators “and”/”or”. Filters related to publication date and language were not used, but the search was limited to randomized clinical trials and humans. Table 1 shows the search strategies used for each database.

### 2.2. Study Screening: Inclusion and Exclusion Criteria

The study selection process was carried out independently by two authors (M.P.-M. and A.O-P.-V), who reviewed all retrieved records by title and abstract. Each study was examined in detail by two reviewers. All disagreements that arose during this phase were resolved by a third author (E.G.-D.). A study was included in our review only if it met all the following inclusion criteria: (I) studies conducted in humans with visceral disorders, as well as functional dyspepsia, gastroesophageal reflux, hiatal hernia, primary sclerosing cholangitis, pancreatitis, gastritis, duodenitis, diabetes, or esophageal stenosis; (II) studies in which transcutaneous or noninvasive stimulation was applied to the VN; and (III) randomized clinical trials, as they provided more quantitative data of interest for conducting the meta-analysis. The exclusion criteria were as follows: (I) studies conducted on animals; (II) studies in which the patient did not receive transcutaneous or noninvasive VNS; and (III) studies other than randomized clinical trials.

### 2.3. Data Extraction

Data extraction was performed independently by two authors (M.P.-M. and E.G.-D.) via a standardized Microsoft Excel form specifically designed for this review, and any discrepancies were resolved through consultation with a third reviewer (A.O.-P.-V.). The extracted information included general study characteristics (authors, year of publication, and study design), the technical parameters of transcutaneous vagus nerve stimulation (stimulation modality [auricular or cervical], frequency, and duration), participant characteristics (type of visceral disorder, sample size, and allocation to intervention or control), and quantitative outcome data, including change-from-baseline or postintervention values, depending on how the outcomes were reported in the original studies, for the outcomes of interest, including gastrointestinal symptoms, cardiac vagal tone (CVT), and cardiac autonomic neuropathy (CAN) (classified as No CAN, Early CAN, or Manifest CAN), among others. When multiple publications originated from the same trial population, all relevant outcomes were extracted; however, only independent datasets were considered for quantitative synthesis to avoid double-counting participants.

For continuous outcomes, mean differences (MDs) or standardized mean differences (SMDs) were extracted depending on the comparability of measurement scales. When standard deviations were not reported, they were calculated from 95% confidence intervals via established statistical transformations. In studies with more than one active stimulation arm (e.g., V10 and V25), each arm was treated as an independent comparison in accordance with Cochrane recommendations, with appropriate adjustment of the shared control group. For crossover trials, quantitative synthesis was performed only when paired outcome data required for an appropriate crossover meta-analysis were available. Otherwise, these studies were included in the qualitative synthesis, and their findings were summarized narratively. Studies reporting only medians with ranges or interquartile ranges, without sufficient information to derive reliable standard deviations, were excluded from the quantitative synthesis. The Supplementary Materials were thoroughly reviewed to ensure the complete and accurate extraction of all the relevant numerical data.

### 2.4. Variables

The primary outcomes analyzed in this systematic review were gastrointestinal symptoms, CVT, systolic blood pressure, and CAN. Gastrointestinal symptoms were assessed using the Gastroparesis Cardinal Symptom Index (GCSI), the Gastrointestinal Symptom Rating Scale (GSRS), the Short-Form Nepean Dyspepsia Index (SF-NDI), and the Dyspepsia Symptom Scale. CVT was evaluated using the CVT index, whereas CAN was classified as No CAN, Early CAN, or Manifest CAN according to the criteria reported in each study. Blood pressure outcomes included systolic blood pressure measurements.

Additional outcomes reported in the included studies, such as pain, anxiety, depression, inflammatory biomarkers, and glycemic parameters (2hPG, FPG, and HbA1c), were also extracted. Pain was assessed using the Visual Analog Scale (VAS), Brief Pain Inventory (BPI), Quantitative Sensory Testing (QST), Capacity of Descending Pain Modulation (CPM), and the Cold Pressor Test. Anxiety and depression were evaluated using the Hamilton Depression Rating Scale (HAM-D), Hamilton Anxiety Rating Scale (HAM-A), and Hospital Anxiety and Depression Scale (HADS). These outcomes were narratively synthesized when quantitative pooling was not feasible.

### 2.5. Assessment of Methodological Quality and Quality of Evidence

First, the methodological quality of the articles included in this review was assessed via the PEDro scale [26]. The PEDro scale consists of eleven items. The first item assesses only external validity and is not included in the final score. Items two through eleven are evaluated on the basis of their presence (scored as one) or absence (scored as zero) in the publication. Thus, the total score for a study can range from zero (minimum) to ten (maximum). Articles scoring fewer than four points are classified as “poor,” those scoring between four and five points as “fair,” those scoring between six and eight points as “good,” and those scoring nine or ten points as “excellent” [27].

In addition, we used the Grading of Recommendations Assessment, Development, and Evaluation (GRADE) approach to assess the certainty of evidence for each meta-analysis [28]. We followed the GRADE checklist proposed by Meader to estimate the quality of evidence, considering the risk of bias in each included study, inconsistency, imprecision, indirectness, and risk of publication bias [29]. Within the GRADE framework, inconsistency was assessed by examining variability in effect estimates and the degree of heterogeneity across the included studies.

However, because fewer than 10 studies were available for each outcome, publication bias could not be formally assessed and was therefore not used as a criterion for downgrading the certainty of evidence. The quality of evidence was classified as high, moderate, low, or very low according to GRADE recommendations. The quality rating for each meta-analysis started at high and was downgraded by one level for each limitation identified. If multiple limitations were present, the overall quality was downgraded by two levels.

Two independent authors (M.P.-M. and A.O.-P.-V.) assessed the risk of bias in the individual studies and the certainty of evidence, with disagreements resolved by a third author (E.G.-D.).

### 2.6. Statistical Analysis

The statistical analysis was performed independently by two authors (M.P.-C. and M.P.-M.) via Review Manager (RevMan 5.4), with all the extracted values cross-checked for accuracy. Continuous outcomes were synthesized using MD when studies used the same measurement scale (e.g., the GSRS) and SMD when the same construct was measured using different scales or metrics, as was the case for CVT.

For trials including more than one active stimulation arm (such as 10 Hz and 25 Hz), each arm was entered as an independent comparison following Cochrane recommendations, adjusting the shared control group when required to avoid unit-of-analysis errors. When standard deviations (SDs) were not provided, they were calculated from 95% confidence intervals via established transformation formulas. Studies reporting only medians and ranges/interquartile ranges, without sufficient information to compute robust SD values, were excluded from the quantitative synthesis and incorporated narratively.

Dichotomous outcomes related to CAN—classified as No CAN, Early CAN, or Manifest CAN—were extracted as event counts. When multiple publications originated from the same trial population, only one independent dataset was considered for evidence synthesis to avoid double-counting participants. Consequently, because only one independent study reported CAN outcomes, these findings were synthesized narratively rather than quantitatively.

Random-effects models were used when clinical or statistical heterogeneity was expected, whereas fixed-effect models were applied when statistical heterogeneity was negligible and the studies were considered sufficiently comparable. Statistical heterogeneity was assessed using the I^2^ statistic and Cochran’s Q test. I^2^ values of approximately 25%, 50%, and 75% were considered indicative of low, moderate, and high heterogeneity, respectively. Because fewer than 10 studies were available for each outcome, publication bias could not be formally assessed; therefore, funnel plots were interpreted descriptively only, and no formal statistical tests (e.g., Egger’s regression test) were performed. All pooled effects were interpreted considering both the statistical significance and the clinical relevance of the magnitude and direction of the observed changes.

## 3. Results

### 3.1. Search Results

**Figure 1:**
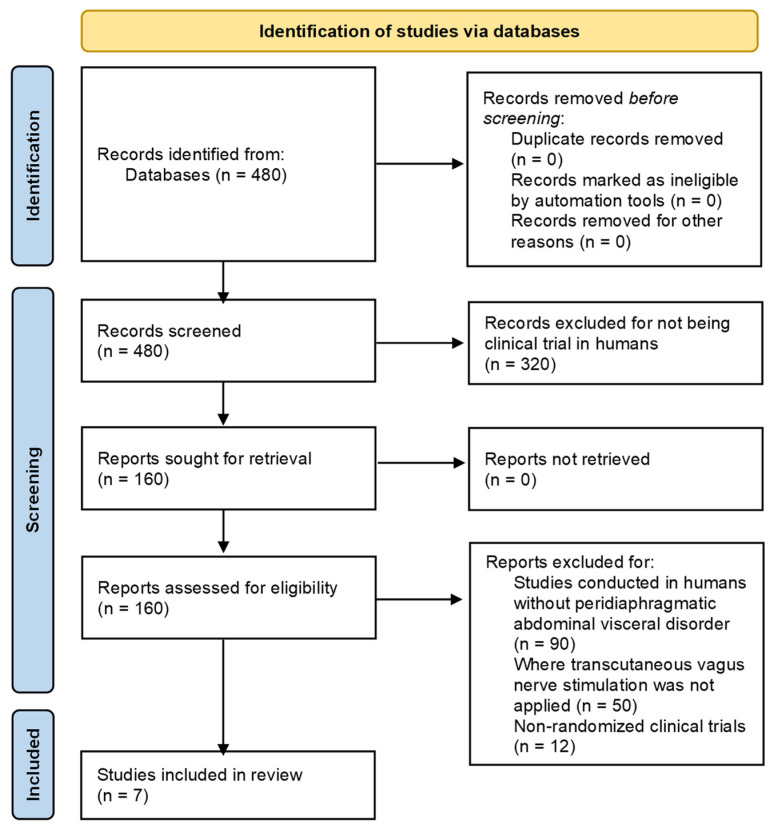
Flow chart diagram of the study selection process (Preferred Reporting Items for Systematic Reviews and Meta-Analyses, PRISMA, guidelines).

A total of 480 records were identified through searches in the following databases: PubMed (n = 83), Scopus (n = 320), and Web of Science (n = 77). Prior to the selection process, database searches were restricted to clinical trials in humans. The remaining articles were carefully assessed to determine their eligibility. As a result, seven articles meeting the predefined inclusion criteria were identified [30,31,32,33,34,35,36]. Figure 1 shows the PRISMA flow diagram corresponding to the study selection process.

### 3.2. Characteristics of the Studies Included in the Review

The seven studies included in this review were randomized trials. In terms of publication year, there was one trial in 2014 [36] and 2017 [35], two trials in 2021 [33,34], and three in 2024 [30,31,32].

These studies provided data from 827 participants. However, two publications [30,31] were secondary reports derived from the same randomized controlled trial and therefore shared the same participant cohort. Consequently, although both publications were included in the qualitative synthesis because they reported different outcomes, only independent datasets were considered in the quantitative synthesis to avoid double-counting participants.

The included studies investigated patients with abdominal visceral disorders, including diabetes or prediabetes (n = 3; 42.86%) [30,31,36], chronic pancreatitis (n = 2; 28.57%) [33,35], and functional dyspepsia (n = 2; 28.57%) [32,34]. Three studies applied cervical tVNS (n = 3; 42.86%) [30,31,33], whereas four used auricular stimulation (n = 4; 57.14%) [32,34,35,36]. All studies compared an experimental group with a control group. Three studies also included a healthy control group (n = 3; 42.86%) [30,34,36]. One study included an additional intervention arm with a different stimulation intensity (n = 1; 14.29%) [32], whereas another employed a crossover design in which participants acted as their own controls (n = 1; 14.29%) [33].

Regarding the evaluated outcomes, three studies assessed gastrointestinal symptoms (n = 3; 42.86%) [31,32,34], blood biomarkers (n = 3; 42.86%) [30,35,36], and CVT (n = 3; 42.86%) [31,33,35]. Two studies evaluated depression and anxiety (n = 2; 28.57%) [32,34], pain (n = 2; 28.57%) [33,35], and CAN (n = 2; 28.57%) [30,31]. Because the crossover trial by Muthulingam et al. [33] did not provide the paired outcome data required for an appropriate crossover meta-analysis, its CVT findings were synthesized narratively. The two publications reporting CAN outcomes originated from the same trial population; therefore, these results were treated as a single independent dataset in the evidence synthesis. Table 2 summarises the characteristics of the included studies.

### 3.3. Methodological Quality

According to the PEDro scale, the mean methodological quality score of the included studies was 9.43/10, indicating overall excellent methodological quality. Five studies included in this meta-analysis reported excellent methodological quality [30,31,32,33,34], whereas two studies were rated as having good methodological quality [35,36]. Overall, most studies fulfilled the criteria related to randomization, allocation concealment, blinding, and reporting of outcomes. The only items that were not consistently satisfied were therapist and assessor blinding in two older studies [35,36], suggesting an overall low risk of bias across the included studies. Table 3 shows the score for each item on the PEDro scale.

### 3.4. Findings in Meta-Analysis

#### 3.4.1. Effect of Transcutaneous Vagus Nerve Stimulation on Gastrointestinal Symptoms

Two studies reporting change-from-baseline GSRS scores were included in the quantitative synthesis, comprising a total of 411 participants. As lower (more negative) change scores indicate a greater reduction in gastrointestinal symptoms, tVNS was associated with significantly greater improvement than sham stimulation, with a pooled mean difference of −0.19 points (95% CI −0.29 to −0.09; *p* = 0.0001).

No statistical heterogeneity was observed (I^2^ = 0%; χ^2^ = 0.28, *p* = 0.59). Although stimulation protocols differed across studies, the direction of the treatment effect was consistent. The individual study estimates favored tVNS, with mean differences of −0.11 (95% CI −0.42 to 0.20) for Kornum et al. [31] and −0.20 (95% CI −0.30 to −0.10) for Shi et al. [32].

**Figure 2:**
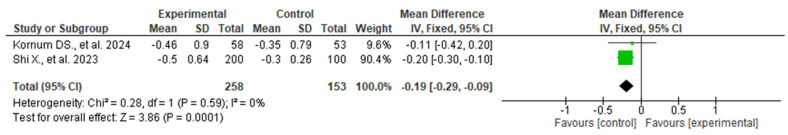
Forest plot of change-from-baseline Gastrointestinal Symptom Rating Scale (GSRS) scores [31,32]. Negative mean differences indicate greater reductions in gastrointestinal symptom severity and therefore favor transcutaneous vagus nerve stimulation.

Overall, the findings indicate that tVNS produces a modest but statistically significant reduction in gastrointestinal symptom severity compared with sham stimulation. However, these results are based on only two studies, and the clinical relevance of the observed effect remains uncertain (Figure 2).

#### 3.4.2. Cardiac Parasympathetic Regulation: CVT

Two studies assessing postintervention CVT were included in the quantitative synthesis, involving a total of 142 participants. A random-effects meta-analysis showed that transcutaneous vagus nerve stimulation did not significantly improve CVT compared with sham stimulation (SMD = 0.19, 95% CI −0.53 to 0.90; *p* = 0.61).

Heterogeneity was substantial (I^2^ = 73%; χ^2^ = 3.74, *p* = 0.05), reflecting variability in the treatment effects across the included studies. Individually, Juel et al. [35] showed a numerically greater CVT in the tVNS group, although the difference was not statistically significant (SMD = 0.60, 95% CI −0.04 to 1.23), whereas Kornum et al. [31] showed a small, nonsignificant effect favouring the control group (SMD = −0.14, 95% CI −0.52 to 0.25).

The crossover trial by Muthulingam et al. [33] was not included in the quantitative synthesis because paired outcome data required for an appropriate crossover meta-analysis were unavailable. Its findings were therefore considered narratively.

**Figure 3:**
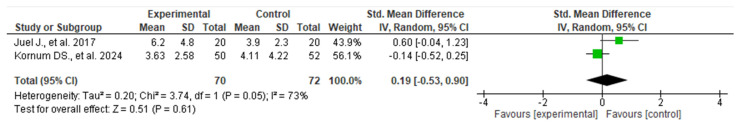
Forest plot of the effect of transcutaneous vagus nerve stimulation on cardiac vagal tone (CVT) [31,35].

Overall, the available evidence indicates that tVNS does not significantly enhance CVT compared with sham stimulation (Figure 3).

#### 3.4.3. Impact of Transcutaneous Vagus Nerve Stimulation on Systolic Blood Pressure

Two studies evaluating postintervention systolic blood pressure, comprising a total of 110 participants, were included in the meta-analysis. Pooled analysis via a fixed-effects model demonstrated that, compared with sham stimulation, transcutaneous vagus nerve stimulation did not significantly modify systolic blood pressure. The combined mean difference was −1.24 mmHg (95% CI −6.69–4.21, *p* = 0.66).

Heterogeneity was absent (I^2^ = 0%; χ^2^ = 0.53, *p* = 0.47), indicating a high degree of consistency between studies. Individually, neither Huang et al. [36] (MD −0.36; 95% CI −5.30–5.58) nor Juel et al. [35] (MD −5.09; 95% CI −19.59–7.79) reported significant differences between groups.

**Figure 4:**
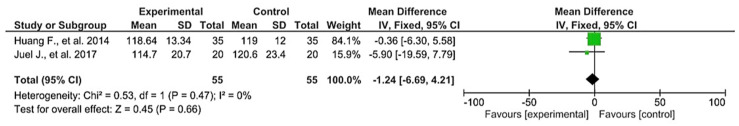
Forest plot of the effect of transcutaneous vagus nerve stimulation on systolic blood pressure [35,36].

Overall, these findings suggest that tVNS does not exert an acute or short-term effect on systolic blood pressure relative to sham stimulation (Figure 4).

#### 3.4.4. Impact of Transcutaneous Vagus Nerve Stimulation on the Prevalence of Cardiac Autonomic Neuropathy

Evidence regarding cardiac autonomic neuropathy (CAN) was available from a single independent randomised controlled trial (n = 131). Although CAN outcomes were reported in two publications [30,31], both analyses were derived from the same participant cohort; therefore, only one independent dataset was considered in order to avoid double-counting participants.

In this trial, the prevalence of CAN (defined as the presence of either Early CAN or Manifest CAN) did not differ between participants receiving transcutaneous vagus nerve stimulation and those receiving sham stimulation (RR 1.14, 95% CI 0.83–1.57).

Overall, the available evidence does not suggest that tVNS modifies the prevalence of CAN. However, this conclusion is based on a single independent study and should therefore be interpreted with caution.

The funnel plots are presented in Supplementary Figures S2–S5. Given the small number of studies available for each outcome (≤3 studies), formal statistical tests for publication bias were not performed, and the funnel plots were interpreted descriptively only.

The certainty of evidence was evaluated using the GRADE approach (Table 4). The certainty of evidence was rated as moderate for gastrointestinal symptoms and systolic blood pressure and as low for CVT and CAN. The main reasons for downgrading were imprecision, inconsistency, and indirectness.

Overall, the available evidence suggests that tVNS may improve gastrointestinal symptoms, whereas no significant effects were observed for CVT, systolic blood pressure, or CAN. However, the certainty of evidence ranged from low to moderate, highlighting the need for further high-quality randomized controlled trials to strengthen confidence in these findings.

## 4. Discussion

This systematic review and meta-analysis synthesized the available evidence on the effects of tVNS in individuals with peridiaphragmatic abdominal visceral disorders. Across the included randomized clinical trials, stimulation protocols varied in frequency, anatomical target (auricular or cervical branches), treatment duration, and patient populations. Despite this variability, the pooled results allow several important observations regarding the physiological and clinical impact of tVNS.

### 4.1. Interpretation of the Main Findings

The most consistent finding of this review was a modest yet statistically significant improvement in gastrointestinal symptoms, as measured by the GSRS. The meta-analysis revealed a small but statistically significant effect in favor of tVNS, with no detectable statistical heterogeneity between the two included studies. These findings suggest that tVNS may improve dyspeptic symptom severity, potentially through the modulation of vagal afferent signaling, improved gastric accommodation, or dampening of neuroimmune activity. Although the effect size is modest, the statistical significance of the findings should not be automatically interpreted as evidence of clinical relevance. Given the limited number of contributing studies (n = 2) and the absence of consistently reported minimal clinically important differences (MCIDs), the clinical significance of this improvement remains uncertain.

In contrast, the pooled analysis of CVT did not reveal significant differences between tVNS and sham stimulation. Individual trials reported mixed and imprecise effects, and substantial heterogeneity was observed. Importantly, the absence of statistically significant pooled effects should not be interpreted as evidence that tVNS lacks autonomic effects. Individual studies reported heterogeneous findings, with Juel et al. [35] reporting a trend towards increased CVT, whereas other studies reported non-significant or mixed effects. These discrepancies may reflect differences in patient populations, stimulation parameters, intervention duration, baseline autonomic status, and methodological approaches used to assess autonomic function. These findings may indicate that short-term neuromodulation is insufficient to produce measurable changes in cardiac parasympathetic regulation, or that CVT metrics may not be sufficiently sensitive to detect subtle autonomic adaptations induced by tVNS. Longer stimulation protocols or alternative autonomic biomarkers may be needed to clarify this physiological relationship.

Similarly, the analysis of systolic blood pressure revealed no significant changes attributable to tVNS. The absence of statistical heterogeneity suggests that the available studies reported similar findings, regardless of patient phenotype or stimulation protocol. Although vagal efferent activation has been proposed to reduce sympathetic activity and improve cardiovascular regulation, the present evidence does not consistently demonstrate an acute hemodynamic effect of tVNS.

Evidence regarding CAN was available from a single independent randomized controlled trial. Although CAN outcomes were reported in two publications, both were derived from the same participant cohort and therefore did not represent independent datasets. The available evidence showed no significant differences in the prevalence of CAN between participants receiving tVNS and those receiving sham stimulation. However, because these findings are based on a single independent study, they should be interpreted with caution and cannot be considered conclusive regarding the effect of tVNS on the prevalence of CAN.

### 4.2. Integration with Vagal Physiology and Previous Evidence

Understanding VN physiology is essential for contextualizing these findings. The VN constitutes a major bidirectional communication system between the body and the brain, contributing to homeostasis [18]. Originating in the brain stem and projecting to the proximal two-thirds of the colon, the VN innervates multiple thoracic and abdominal viscera. It is a mixed nerve composed of approximately 20% efferent and 80% afferent fibers [37], meaning that peripheral stimuli—especially auricular stimulation—can induce significant central effects through projections to the solitary tract nucleus, locus coeruleus, and limbic structures [18,38].

TVNS has emerged as a noninvasive alternative to traditional invasive VNS, with comparable physiological effects [39]. This technique targets cutaneous branches of the VN, either in the outer ear through the auricular branch or in the neck through the cervical branch, thereby avoiding surgical implantation and enabling wider clinical application [40]. The studies included in this review employed both cervical and auricular modalities. Cervical tVNS likely stimulates both afferent and efferent fibers of the cervical branch [41,42,43]. Moreover, activation of vagal fibers projecting to the laryngeal and pharyngeal muscles near the stimulation site [44] may induce antidromic signaling [45]. In contrast, auricular tVNS specifically targets the auricular branch of the VN, also known as Arnold’s nerve [38,46,47]. This technique is more recent [48] and has been proposed as a therapeutic strategy for epilepsy [49], depression [50], migraine [51], and tinnitus [52]. Subsequent research has also identified several reflexes and phenomena suggesting functional connectivity between the external ear and internal viscera [53,54]. The rationale for taVNS therefore relies on the cutaneous representation of the auricular branch and its central projections to autonomic regulatory structures [18,38].

Although both forms of tVNS exhibit strong physiological plausibility, the heterogeneous findings of this review indicate that their clinical effects may not be generalizable across all abdominal visceral disorders. Treatment responsiveness may differ according to the underlying disease, as conditions such as functional dyspepsia, diabetes-related autonomic dysfunction, and chronic pancreatitis involve distinct pathophysiological mechanisms and varying degrees of autonomic impairment. Therefore, disease-specific mechanisms, patient characteristics, stimulation parameters, and treatment duration likely modulate therapeutic outcomes.

The inclusion of both cervical and auricular stimulation modalities may also have contributed to the observed clinical heterogeneity. Formal subgroup analyses according to stimulation modality were not feasible because of the limited number of studies available for each outcome. Nevertheless, some modality-related differences warrant consideration. Auricular tVNS primarily stimulates afferent fibers of the auricular branch of the VN, promoting central autonomic modulation through brain stem projections [18,38,46,47], whereas cervical tVNS may activate both afferent and efferent vagal pathways [41,42,43,44,45]. Interestingly, Juel et al. [35] reported a trend towards increased CVT following auricular stimulation, whereas the cervical stimulation studies included in the quantitative synthesis did not demonstrate significant improvements in CVT [30,31]. Although these findings should be interpreted cautiously, they raise the possibility that the stimulation site may influence autonomic responses and could partly contribute to the heterogeneity observed across studies.

Finally, the absence of consistent measurable autonomic or cardiovascular changes, despite the strong physiological rationale, suggests that stimulation intensity, treatment duration, or the selected biomarkers may not adequately capture the central autonomic effects induced by tVNS. Autonomic regulation was evaluated using a limited set of cardiovascular markers, primarily CVT, systolic blood pressure, and CAN. Although clinically relevant, these measures do not fully reflect the complexity of autonomic nervous system function. Consequently, the present findings should be interpreted as reflecting selected aspects of cardiovascular autonomic regulation rather than autonomic regulation as a whole.

### 4.3. Psychological Outcomes and the Brain–Gut Axis

Although anxiety and depression outcomes could not be included in the quantitative synthesis because the available studies reported these variables as medians and interquartile ranges, the reported findings deserve consideration within the context of the brain–gut axis. Two included studies evaluated psychological outcomes following auricular tVNS in patients with functional dyspepsia [32,34].

Shi et al. [32] reported significant reductions in both anxiety and depression scores after four weeks of treatment, with greater improvements observed in the active stimulation groups than in the sham group. Similarly, Zhu et al. [34] found significant decreases in anxiety and depression scores after two weeks of taVNS, whereas no improvements were observed following sham stimulation. These findings suggest that tVNS may improve psychological outcomes in patients with functional dyspepsia, in addition to alleviating gastrointestinal symptoms.

The observed psychological improvements are consistent with the physiological role of the vagus nerve within the brain–gut axis. Vagal afferent pathways project to the solitary tract nucleus and subsequently influence brain regions involved in emotional regulation, including limbic and autonomic networks [18,38]. Given the close relationship between gastrointestinal symptoms, anxiety, and depression in functional gastrointestinal disorders, improvements in psychological well-being may contribute, at least partially, to the symptomatic benefits observed after tVNS.

Nevertheless, these findings should be interpreted cautiously because quantitative pooling was not possible, and only two studies reported relevant psychological outcomes. Furthermore, both studies were conducted in patients with functional dyspepsia; therefore, these findings cannot be readily generalized to other abdominal visceral disorders. Future trials should provide complete parametric data to facilitate quantitative synthesis and improve understanding of the interaction between psychological and gastrointestinal responses to tVNS.

### 4.4. Relationships with Other Noninvasive Neuromodulation Strategies

Situating tVNS within the broader context of noninvasive neuromodulation also highlights complementary physiological mechanisms that may contribute to symptom modulation. Breathing practices, for example, increase parasympathetic activity as reflected in heart rate variability measurements [55], and deep breathing has been shown to reduce anxiety and stress levels [56]. Among individuals with diabetes, slow and deep diaphragmatic breathing may lower glycated hemoglobin levels and blood pressure, likely through enhanced parasympathetic activation [57,58]. Given their shared parasympathetic mechanisms, combining tVNS with structured breathing interventions may represent a promising area for future investigation.

Deep respiration also activates the diaphragm, which receives both motor and sensory innervation from the phrenic nerve [59]. Within the thoracic cavity, this phrenic nerve supplies the mediastinal and diaphragmatic pleura, the right atrium, the fibrous pericardium, and the parietal pericardium [60,61]. Consequently, recent studies have explored transcutaneous phrenic nerve stimulation in patients with abdominal visceral disorders [62,63]. Preliminary evidence also suggests that transcutaneous phrenic nerve stimulation may have therapeutic potential in abdominal visceral disorders. For example, Pérez-Montalbán et al. [64] reported significant improvements in the Neck Disability Index, VAS scores, Central Sensitization Inventory scores, cervical mobility, and quality of life in individuals with inflammatory bowel disease and neck pain. Although these findings require confirmation in larger clinical trials, they support the concept that noninvasive neuromodulation targeting peripheral nerves may offer complementary therapeutic approaches for patients with abdominal visceral disorders.

### 4.5. Limitations

Several limitations should be considered when these findings are interpreted. First, the number of available randomized controlled trials remains limited, and most studies included relatively small sample sizes. In addition, stimulation parameters (including site, intensity, frequency, and duration) varied considerably across studies, which may have diluted intervention-specific effects.

Second, substantial clinical heterogeneity existed across the included populations. The studies evaluated patients with different gastrointestinal and autonomic conditions, including functional dyspepsia, diabetes/prediabetes, chronic pancreatitis, and other visceral disorders. These conditions differ in their underlying pathophysiological mechanisms and degree of autonomic dysfunction, which may have influenced responsiveness to transcutaneous vagus nerve stimulation and limited the generalizability of the pooled estimates across all gastrointestinal disorders.

Third, both auricular and cervical transcutaneous vagus nerve stimulation modalities were included in the quantitative synthesis. Although both approaches aim to modulate vagal activity, they differ in their anatomical targets, peripheral pathways, and potentially their mechanisms of action. The limited number of available studies precluded meaningful subgroup analyses according to stimulation modality, making it difficult to determine whether the stimulation site contributed to differences in clinical or autonomic outcomes. Therefore, modality-specific effects cannot be excluded, and future studies directly comparing auricular and cervical tVNS are warranted.

A relevant limitation concerns the analysis of psychological outcomes. Although some studies have assessed variables such as anxiety and depression, these variables are frequently reported as medians and interquartile ranges without sufficient dispersion data to estimate standard deviations. Consequently, these outcomes could not be included in the quantitative synthesis, despite their potential relevance to autonomic regulation and the brain–gut axis.

Moreover, autonomic regulation was evaluated using a limited number of cardiovascular markers, primarily CVT, systolic blood pressure, and CAN. These measures do not fully capture the complexity of autonomic nervous system function, and therefore the findings should not be interpreted as reflecting all potential autonomic effects of tVNS.

Finally, evidence regarding CAN was available from only a single independent randomized controlled trial. Although CAN outcomes were reported in two publications, both were derived from the same participant cohort and therefore could not be quantitatively pooled. Consequently, conclusions regarding the effects of tVNS on CAN remain limited. Similarly, the quantitative synthesis of CVT excluded one crossover trial because paired outcome data required for an appropriate crossover meta-analysis were unavailable, further reducing the amount of evidence available for this outcome. In addition, the relatively short duration of most interventions restricts conclusions regarding long-term autonomic, cardiovascular, or inflammatory adaptations.

### 4.6. Clinical Implications

The findings of this review suggest that tVNS may represent a promising noninvasive adjunctive neuromodulatory intervention capable of providing modest improvements in gastrointestinal symptoms, particularly in functional dyspepsia. Its favorable safety profile, ease of application, and accessibility make it an attractive complementary therapeutic option. However, the observed benefit was based on only two independent studies, and the clinical relevance of the pooled effect remains uncertain.

Clinicians should adopt a cautious interpretation regarding its broader physiological effects. Current evidence does not consistently demonstrate improvements in the cardiovascular autonomic markers assessed in this review. No statistically significant pooled effects were observed for CVT or systolic blood pressure, whereas evidence regarding CAN was limited to a single independent randomized controlled trial. Furthermore, these markers represent only selected aspects of autonomic regulation and should not be interpreted as reflecting autonomic nervous system function as a whole. Therefore, tVNS should currently be considered primarily as a symptomatic intervention rather than a strategy for restoring global autonomic function.

Future research may explore the integration of tVNS with other vagotonic interventions, such as slow diaphragmatic breathing, which has demonstrated beneficial effects on autonomic regulation and cardiovascular function. Such combined approaches warrant further investigation to determine whether complementary mechanisms translate into improved clinical outcomes. Future studies should also evaluate longer treatment durations, standardized stimulation protocols, and disease-specific populations to better define the clinical role of tVNS in abdominal visceral disorders.

## 5. Conclusions

This systematic review and meta-analysis demonstrated that transcutaneous vagus nerve stimulation may provide modest but statistically significant improvements in gastrointestinal symptoms in individuals with peridiaphragmatic abdominal visceral disorders. However, the available evidence for this outcome was based on only two studies, and the clinical relevance of the observed effect remains uncertain.

No statistically significant pooled effects were observed for CVT or systolic blood pressure. Evidence regarding CAN was limited to a single independent randomized controlled trial, which did not demonstrate a significant effect of tVNS on the prevalence of CAN. Nevertheless, the available evidence remains limited and heterogeneous and should not be interpreted as demonstrating the absence of autonomic effects. Rather, current findings suggest that the autonomic effects of tVNS remain incompletely understood and require further investigation.

Overall, tVNS appears to be a promising symptomatic neuromodulatory intervention, particularly in dyspeptic conditions, although the current evidence supports its use primarily for symptom relief rather than for the restoration of cardiovascular autonomic function.

Further high-quality randomized controlled trials with standardized stimulation protocols, larger sample sizes, longer follow-up periods, and more comprehensive autonomic outcome measures are needed to better define the therapeutic role of tVNS. Improved reporting of psychological and autonomic outcomes will also be essential to enable more robust future evidence synthesis.

## Figures and Tables

**Table 1:** Search strategy used in each database.

Databases	Search Strategy	Limits
PubMed	((transcutaneous vagal nerve stimulation OR noninvasive vagal nerve stimulation OR transcutaneous auricular vagal nerve stimulation) AND (peridiaphragmatic abdominal viscera disorder OR functional dyspepsia OR gastroesophageal reflux OR hiatal hernia OR primary sclerosing cholangitis OR pancreatitis OR gastritis OR duodenitis OR diabetes OR esophageal stenosis))	Clinical Trial AND Humans
Scopus	ALL FIELDS (((“transcutaneous vagal nerve stimulation” OR “non-invasive vagal nerve stimulation” OR “transcutaneous auricular vagal nerve stimulation”) AND (“peridiaphragmatic abdominal viscera disorder” OR “functional dyspepsia” OR “gastroesophageal reflux” OR “hiatal hernia” OR “primary sclerosing cholangitis” OR “pancreatitis” OR “gastritis” OR “duodenitis” OR “diabetes” OR “esophageal stenosis”)))	Article AND Humans
Web of Science	TOPIC ((transcutaneous vagal nerve stimulation OR non-invasive vagal nerve stimulation OR transcutaneous auricular vagal nerve stimulation) AND (peridiaphragmatic abdominal viscera disorder OR functional dyspepsia OR gastroesophageal reflux OR hiatal hernia OR primary sclerosing cholangitis OR pancreatitis OR gastritis OR duodenitis OR diabetes OR esophageal stenosis))	Clinical Trial AND Humans

**Table 2:** Characteristics of the studies included in the systematic review and meta-analysis.

Studies	Vagus Nerve Stimulation	Frequency	Duration	Visceral Disorder	Group Distribution	Sample	Variables	Quantitative Data
Okdahl T., et al. 2024 [30]	Transcutaneous cervical vagus nerve stimulation	25 Hz 60 mA	2 min -Period 1: 4 times a day, both sides, 7 days -Period 2: 2 times a day, on both sides, 56 days	Diabetes	-Stimulation -Sham stimulation -Control	131 started 116 finished +40 control	-Plasma concentrations of inflammatory cytokines -CAN	No differences in delta concentrations of inflammatory cytokines between active and sham tVNS in either study period. The change in TNF-α levels between CAN status (no CAN versus early/manifest CAN) during active treatment approached statistical significance −0.11 (−0.28–0.05) vs. 0.06 (−0.04–0.16) (*p* = 0.052).
Kornum DS., et al. 2024 [31]	Transcutaneous cervical vagus nerve stimulation	25 Hz 60 mA	2 min -Period 1: 4 times a day, both sides, 7 days -Period 2: 2 times a day, on both sides, 56 days	Diabetes	-Stimulation -Sham stimulation	131 started 116 finished	-GCSI -GSRS -CAN -CVT	No statistically significant differences in GCSI or GSRS responses were observed between active and sham stimulation in either study period. No differences were seen in the CAN score, except for the inspiration/expiration ratio in study period 1, where the sham group showed a higher median decrease (−0.02; IQR −0.06 to 0.02) than the active group (−0.01; IQR −0.02 to 0.03) (*p* = 0.02). Neither acute nor long-term changes in CVT were observed.
Shi X., et al. 2024 [32]	Transcutaneous auricular vagus nerve stimulation	Groups: V10–10 Hz V25–25 Hz 0.5–1.5 mA	30 min 30 s on/ 30 s off 2 times a day 4 weeks	Functional dyspepsia	-Stimulation V10 -Stimulation V25 -Sham stimulation	300 started 284 finished	-GSRS -SF-NDI -HAMD -HAMA	After 4 weeks of treatment, both the V10 and V25 groups showed a significantly greater reduction in stomach pain and bloating compared with the sham group (all *p* < 0.05). The decreases in GSRS (β −0.2, 95% CI −0.3 to −0.1, *p* < 0.001; β −0.2, 95% CI −0.3 to −0.1, *p* < 0.001), SF-NDI (β −2.5, 95% CI −4.4 to −0.6, p0.009; β −2.4, 95% CI −4.3 to −0.6, *p* = 0.012), HAMD (β −2.1, 95% CI −3.0 to −1.2, *p* < 0.001; β −1.9, 95% CI −2.9 to −1.0, *p* < 0.001), and HAMA (β −1.5, 95%CI −2.3 to −0.6, *p* = 0.001; β −1.7, 95% CI −1.1 to −0.9, *p* < 0.001) scores in both taVNS groups were higher than the sham group.
Muthulingam JA., et al. 2021 [33]	Transcutaneous cervical vagus nerve stimulation	25 Hz	2 min 3 times a day, on both sides, 2 weeks	Chronic pancreatitis	-Stimulation and sham stimulation. Crossed	28 started 16 finished	-VAS -BPI -CVT	A significant reduction was found in the average pain scores (−0.7 ± 1.2, 95%CI −1.31–0.003), (*p* = 0.049) and the maximal pain score (−1.3 ± 1.7, 95%CI −2.21–0.42), (*p* = 0.007) comparing tVNS treatment with baseline. Also, significant reductions were detected for sham treatment as compared with baseline. Compared to sham, tVNS treatment induced a decrease in heart rate (−3.7 BPM, 95% CI −6.7–0.6) (*p* = 0.02), while no change in the CVT was observed (*p* = 0.18).
Zhu Y., et al. 2021 [34]	Transcutaneous auricular vagus nerve stimulation	25 Hz 0.5–1.5 mA	60 min 2 s on/ 3 s off 2 times a day, 2 weeks	Functional dyspepsia	-Stimulation -Sham stimulation -Control	36 + 39 controls	-Dyspepsia Symptom Scale -HADS -Assessment of autonomic functions	TaVNS was effective in reducing the dyspeptic symptom assessed by questionnaire compared with the baseline [8.0 (0.0, 28.0) vs. 10.0 (4.0, 34.0), *p* = 0.010, n = 18]. The 2-week taVNS treatment significantly reduced both anxiety [5.5 (1.0, 14.0) vs. 8.0 (4.0, 16.0), *p* = 0.002] and depression scores [2.5 (0.0, 8.0) vs. 5.0 (1.0, 12.0), *p* < 0.001]. However, no improvement was noted with sham stimulation. Vagal activity (HF) was lower in the patients with functional dyspepsia in comparison with the HC in fasting state, and the 2-wk taVNS, but not the sham group, increased HF in both fasting and postprandial states.
Juel J., et al. 2017 [35]	Transcutaneous auricular vagus nerve stimulation	30 Hz 0.1–10 mA	60 min 1 day	Chronic pancreatitis	-Stimulation and breathing protocol -Sham stimulation and sham breathing protocol	20	-Blood pressure -CVT -QST -CPM	Compared to sham stimulation, an increase in CVT was seen after tVNS (3.9 ± 2.3 vs. 6.2 ± 4.8; *p* = 0.02). Furthermore, the mean blood pressure was significantly lowered during tVNS compared to baseline (86.4 ± 15.5 mmHg vs. 81.6 ± 16.0 mmHg; *p* = 0.046), while no significant effect on diastolic blood pressure was observed. TVNS induced no demonstrable differences in muscle pressure thresholds or bone pressure thresholds compared to sham stimulation. A diminished CPM response was seen after tVNS when compared to sham stimulation (7.6 ± 22.5% vs. 26.6 ± 18.8%; *p* = 0.02).
Huang F., et al. 2014 [36]	Transcutaneous auricular vagus nerve stimulation	20 Hz 1 mA	20 min 2 times a day, on both sides, 12 weeks	Prediabetes (glucose tolerance)	-Stimulation -Sham stimulation -Control	102	-Change in 2hPG levels measured -Changes in FPG and HbA1c levels -Blood pressure	A significant difference in 2hPG between groups was observed over the course of the experiment (F(2) = 5.79, *p* = 0.004). The decrease in 2hPG was significantly greater in the taVNS group compared to that in the sham taVNS group. Further analysis of other secondary outcomes indicated that the taVNS and sham taVNS groups differed significantly in systolic blood pressure over time (F(1) = 4.21, *p* = 0.044). In the taVNS group, systolic blood pressure dropped from 123.69 ± 14.14 (mean ± SD) to 118.64 ± 13.34, while in the sham taVNS group, systolic blood pressure remained at 119 ± 12. Repeated measures ANOVA between the taVNS and no-treatment control indicated significant differences in FPG (F(2) = 10.62, *p* < 0.001), 2hPG (F(2) = 25.18, *p* < 0.001) and HbA1c (F(1) = 12.79, *p* = 0.001) between groups over the course of the 12 weeks.

CAN, Cardiac Autonomic Neuropathy; tVNS, transcutaneous vagus nerve stimulation; GCSI, Gastroparesis Cardinal Symptom Index; GSRS, Gastrointestinal Symptom Rating Scale; CVT, Cardiac Vagal Tone; SF-NDI, Short-Form Nepean Dyspepsia Index; HAMD, Hamilton Depression Scale; HAMA, Hamilton Anxiety Scale; taVNS, transcutaneous auricular vagus nerve stimulation; VAS, Visual Analog Scale; BPI, Brief Pain Inventory; HADS, Hospital Anxiety and Depression Scale; QST, Muscle and Bone Pain Pressure Thresholds; CPM, Capacity of Descending Pain Modulation.

**Table 3:** Results of methodological quality and risk of bias on the PEDro scale.

Study	1	2	3	4	5	6	7	8	9	10	11
Okdahl T., et al. 2024 [30]	YES	YES	YES	YES	YES	YES	YES	YES	YES	YES	YES
Kornum DS., et al. 2024 [31]	YES	YES	YES	YES	YES	YES	YES	YES	YES	YES	YES
Shi X., et al. 2024 [32]	YES	YES	YES	YES	YES	YES	YES	YES	YES	YES	YES
Muthulingam JA., et al. 2021 [33]	YES	YES	YES	YES	YES	YES	YES	YES	YES	YES	YES
Zhu Y., et al. 2021 [34]	YES	YES	YES	YES	YES	YES	YES	YES	YES	YES	YES
Juel J., et al. 2017 [35]	YES	YES	YES	YES	YES	NO	NO	YES	YES	YES	YES
Huang F., et al. 2014 [36]	YES	YES	YES	YES	YES	NO	NO	YES	YES	YES	YES

Abbreviations: PEDro, Physiotherapy Evidence Database. PEDro items: (1) eligibility criteria; (2) random allocation; (3) concealed allocation; (4) baseline comparability; (5) participant blinding; (6) therapist blinding; (7) assessor blinding; (8) adequate follow-up; (9) intention-to-treat analysis; (10) between-group comparisons; and (11) point estimates and variability.

**Table 4:** Certainty of evidence according to the GRADE approach.

Outcome	Studies (RCTs)	Participants	Effect Estimate (95% CI)	Certainty of Evidence
Gastrointestinal symptoms	2	411	MD −0.19 (−0.29 to −0.09)	⨁⨁⨁◯ Moderate
Cardiac vagal tone (CVT)	2	142	SMD 0.19 (−0.53 to 0.90)	⨁⨁◯◯ Low
Systolic blood pressure	2	110	MD −1.24 (−6.69 to 4.21)	⨁⨁⨁◯ Moderate
Cardiac autonomic neuropathy (CAN)	1	131	RR 1.14 (0.83 to 1.57)	⨁⨁◯◯ Low

Footnote: The certainty of evidence was assessed using the GRADE approach. Evidence was downgraded due to imprecision (gastrointestinal symptoms and systolic blood pressure), inconsistency and imprecision (CVT), and indirectness and imprecision (CAN). The certainty of evidence was assessed using the GRADE approach. Certainty ratings are represented as follows: ⊕⊕⊕◯ = moderate certainty; ⊕⊕◯◯ = low certainty; filled circles (⊕) indicate the level of confidence in the evidence, whereas empty circles (◯) indicate downgrading of certainty.

## Data Availability

All the materials and data generated can be found in the article.

## Supplementary Materials

The following supporting information can be downloaded at: https://www.mdpi.com/article/10.3390/neurolint18070127/s1, Figure S1: PRISMA checklist, Figure S2: Funnel plot cardiac vagal tone, Figure S3: Funnel plot gastrointestinal symptoms, Figure S4: Funnel plot cardiac autonomic neuropathy, Figure S5: Funnel plot systolic blood pressure.
