# Supplementary figures and images for: Effects of Transcutaneous Vagus Nerve Stimulation on Gastrointestinal Symptoms and Cardiovascular Autonomic Outcomes: A Systematic Review and Meta-Analysis
Source: Neurol Int. 2026 Jul 3;18(7):127. doi: 10.3390/neurolint18070127 (PMC13414916; doi:10.3390/neurolint18070127)

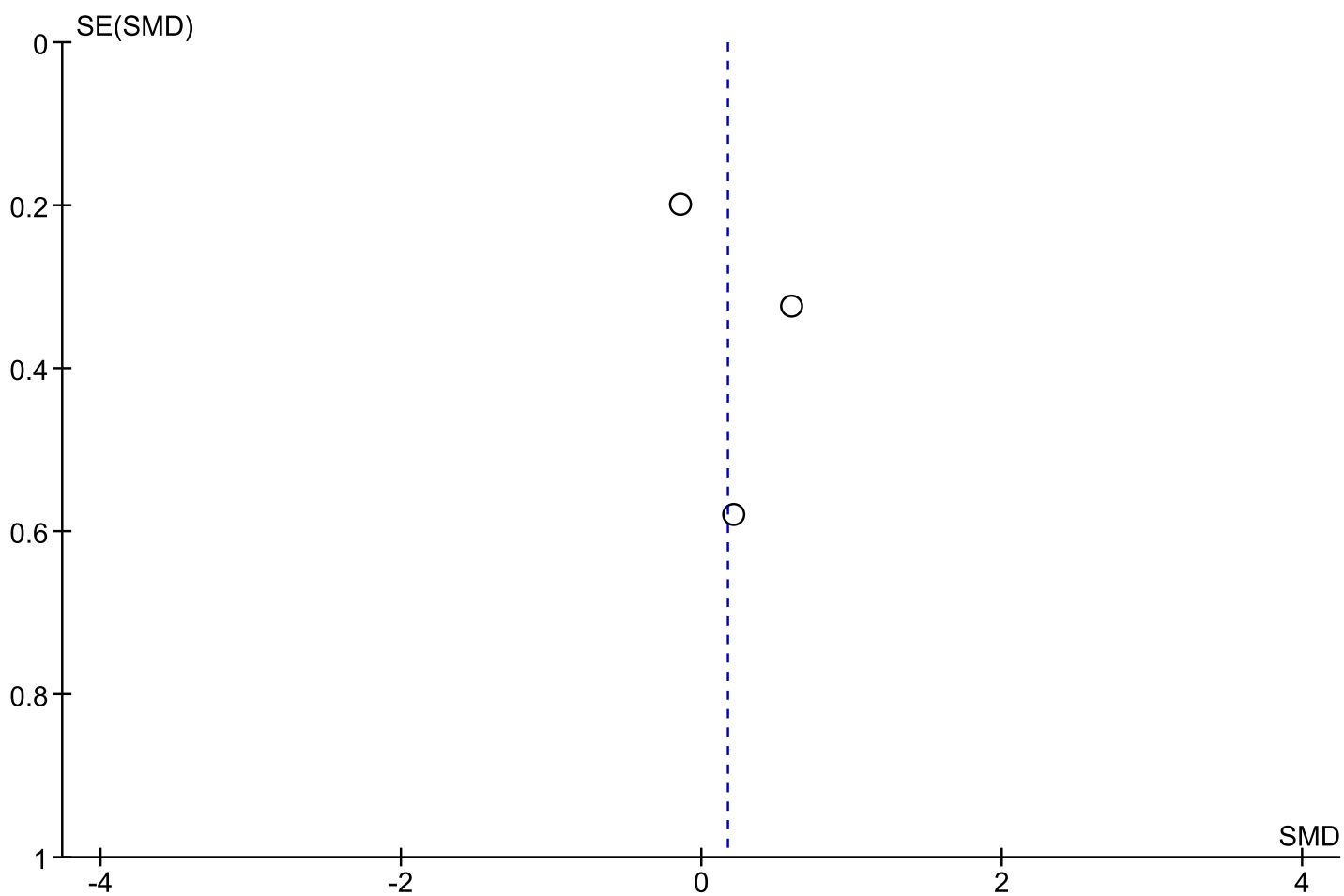

Supplement: Supplementary file 1 [file neurolint-18-00127-s001.zip › Figure S2. Funnel plot cardiac vagal tone (CVT).pdf]

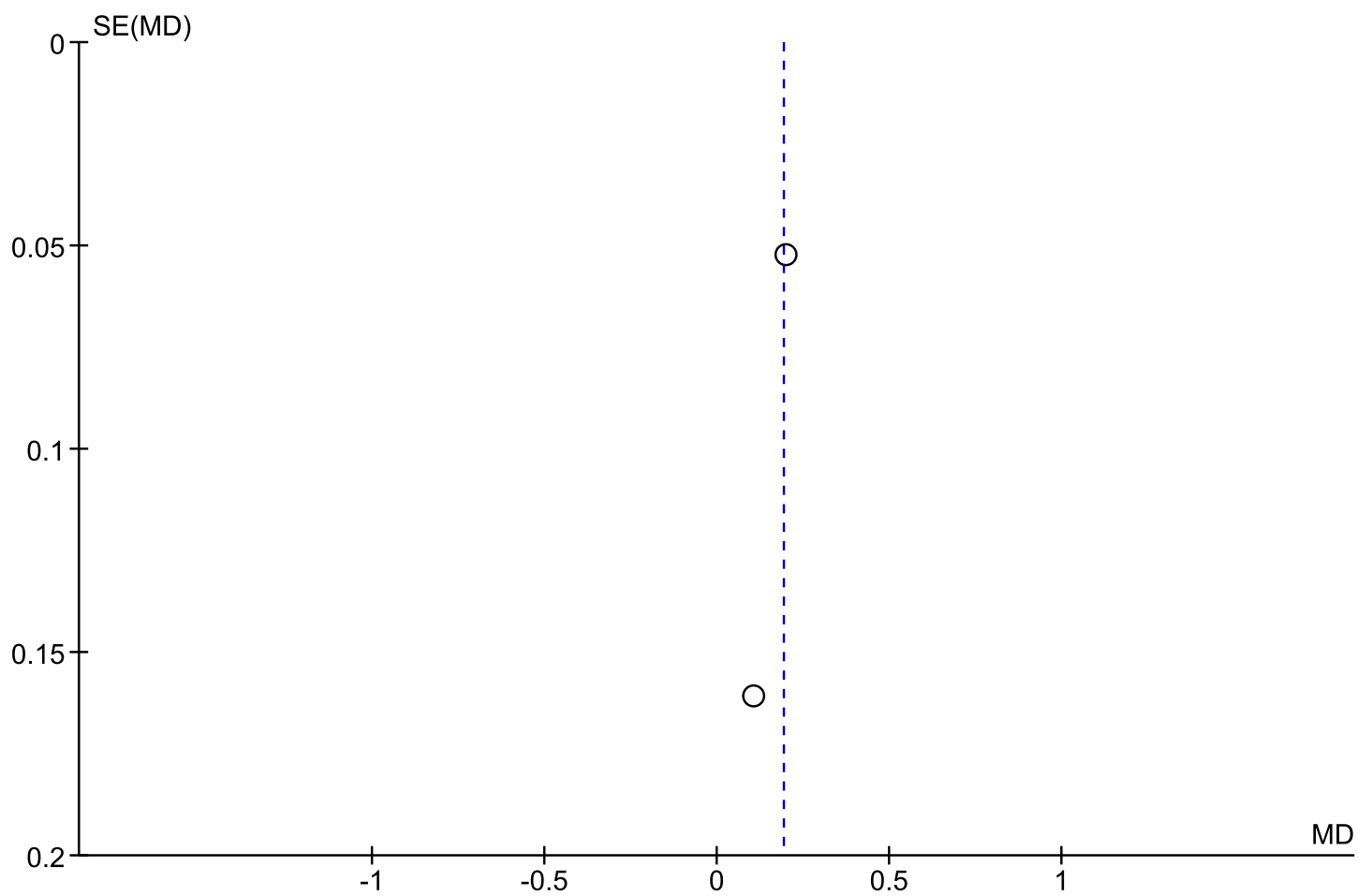

Supplement: Supplementary file 1 [file neurolint-18-00127-s001.zip › Figure S3. Funnel plot gastrointestinal symptoms.pdf]

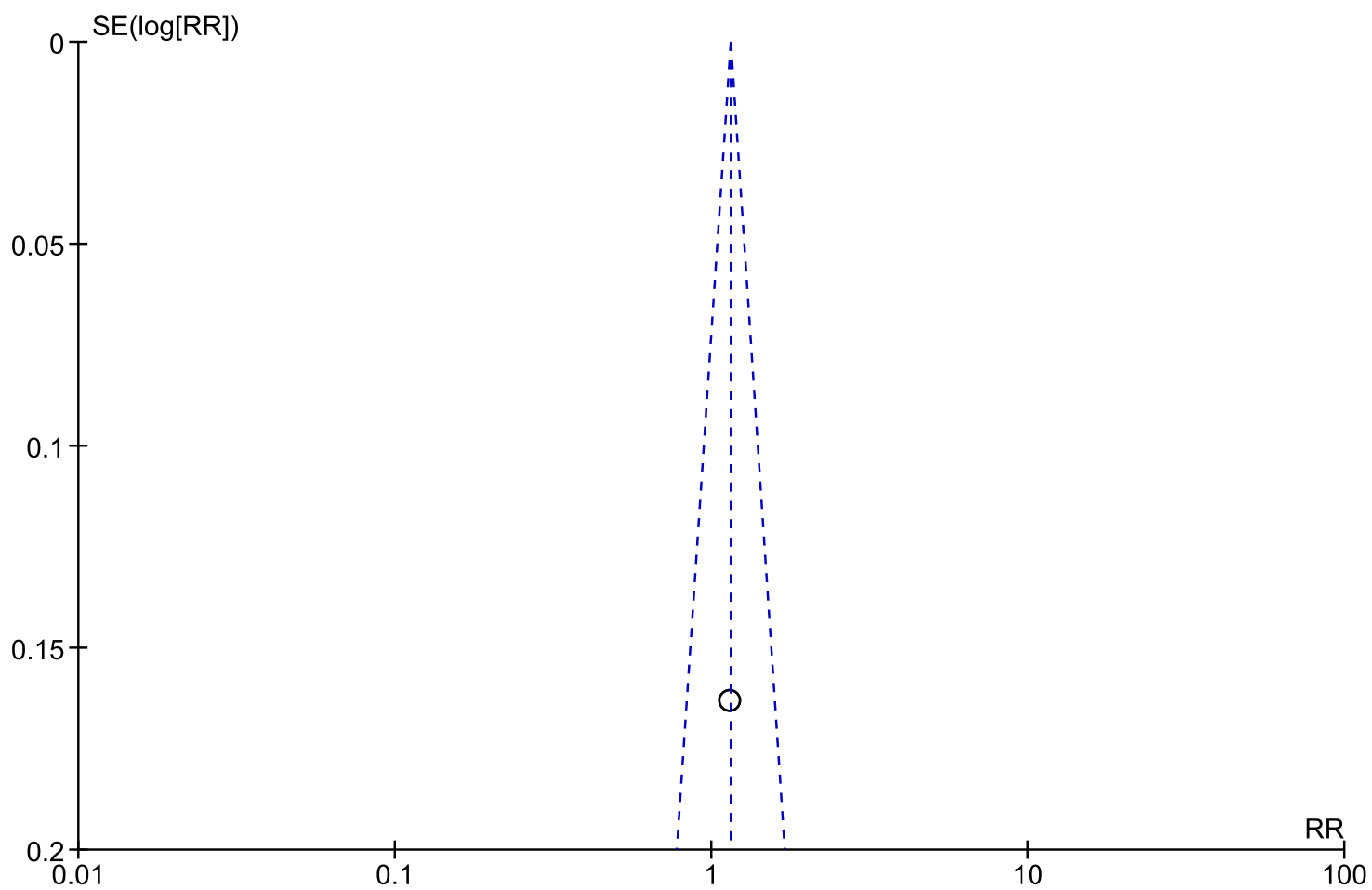

Supplement: Supplementary file 1 [file neurolint-18-00127-s001.zip › Figure S4. Funnel plot cardiac autonomic neuropathy (CAN).pdf]

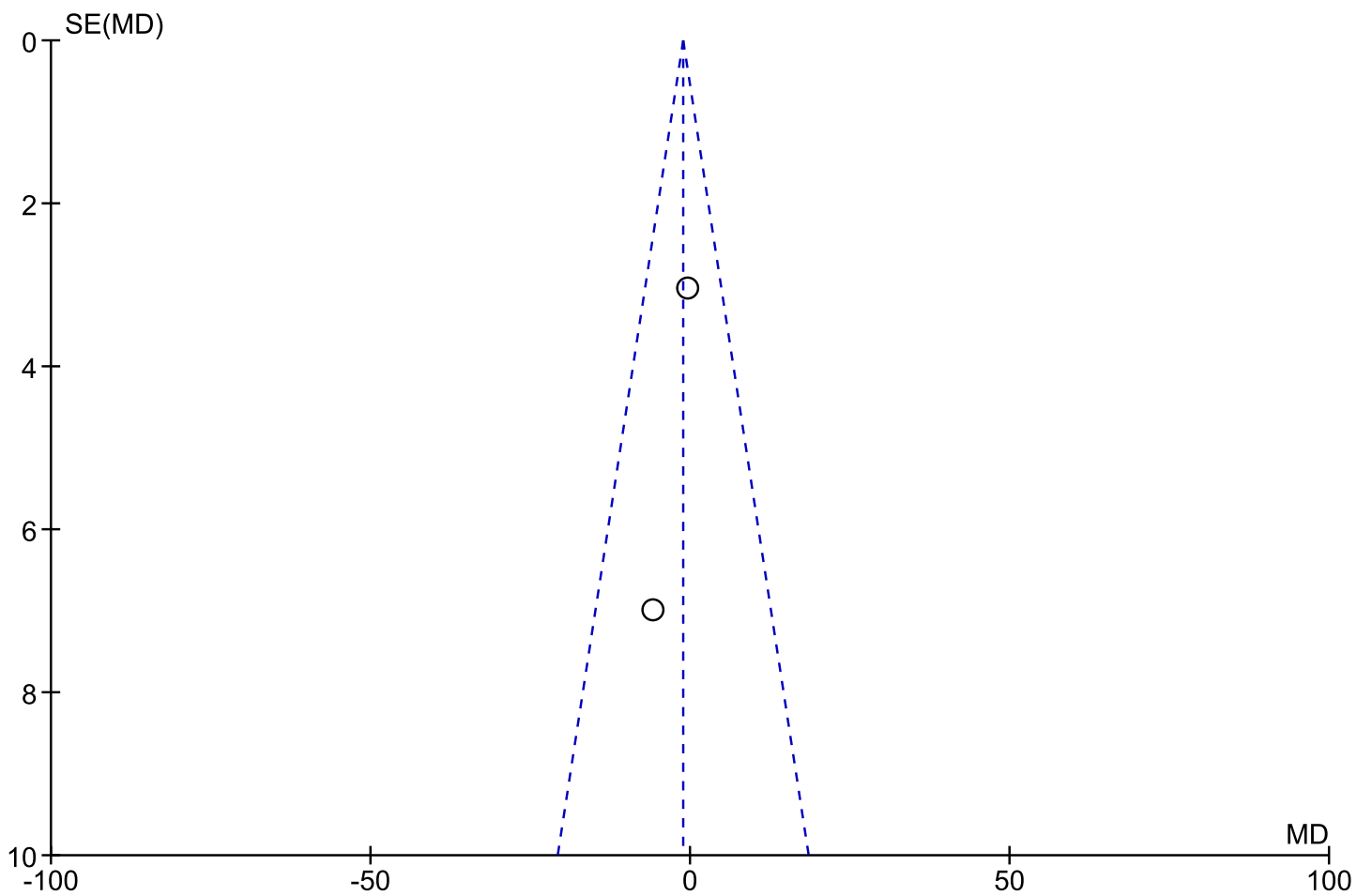

Supplement: Supplementary file 1 [file neurolint-18-00127-s001.zip › Figure S5. Funnel plot systolic blood pressure.pdf]
